# Amyand’s hernia: a case report

**DOI:** 10.1093/jscr/rjaf140

**Published:** 2025-04-03

**Authors:** India Plath, Michael Auld

**Affiliations:** Department of General Surgery, Ipswich Hospital, Chelmsford Avenue, Ipswich, Queensland 4305, Australia; Department of General Surgery, Ipswich Hospital, Chelmsford Avenue, Ipswich, Queensland 4305, Australia

**Keywords:** Amyand’s hernia, appendicitis, inguinal, laparoscopic

## Abstract

An Amyand’s hernia is a type of inguinal hernia where the appendix protrudes into the hernial sac. Pre-operative diagnosis is clinically challenging and ultimately the diagnosis is made intra-operatively. We report on a 75-year-old lady who presented with abdominal pain and a lump in her right groin region. She proceeded to theatre and had a diagnostic laparoscopy where she was diagnosed with an Amyand’s hernia. The appendix was mildly inflamed and there was an ischemic appearance of the mesoappendix. According to the guidelines proposed by Losanoff and Basson she subsequently underwent a successful laparoscopic appendicectomy and primary repair of the hernia.

## Introduction

Inguinal hernias are a common surgical presentation whereby abdominal organs and associated contents protrude through the inguinal canal. Generally, the sac of an inguinal hernia will contain omentum or intraabdominal contents. A rare phenomenon that occurs in approximately 1% of inguinal hernias is the protrusion of the appendix into the sac [1]. This is known as Amyand’s hernia [1, 2]. Amyand’s hernia was first described by Claudius Amyand in 1735 when an 11-year-old boy presented with an appendix in his right inguinal hernia sac and underwent a successful appendicectomy [3]. Clinically, the appendix in an Amyand’s hernia can present from normal tissue to appendicitis to associated complications such as perforation [4].

Preoperative diagnosis of an Amyand’s hernia is challenging due to the absence of specific clinical examination findings. Common symptoms of an inguinal hernia include tenderness in the groin region with an associated mass. Imaging, if any, is often ordered to exclude potential complications of inguinal hernia, such as bowel obstruction or perforation. Imaging can assist in diagnosis of Amyand’s hernia however, the definitive diagnosis of Amyand’s hernia is made intra-operatively [2, 4].

A classification proposed by Losanoff and Basson of the appearance of the appendix in addition to secondary features helps to guide the surgical management of Amyand’s hernia [1, 5].

## Case report

A 75-year-old woman presented to the emergency department following two days of a dull ache in her lower right abdomen. She was in the process of moving house, which involved carrying heavy boxes, and thus attributed the pain to myalgias. She had noticed a small mass in her right lower abdomen that was tender to palpate on the second day of pain. This pain subsequently worsened, prompting her presentation. The patient had normal bowel movements and denied subjective fevers, nausea, or vomiting. Past medical history included hypertension and mild asthma, for which she did not use regular bronchodilators. Her past surgical history included two previous caesarean sections over 30 years prior (Pfannensteil incision) and a subsequent lower midline hysterectomy.

**Figure 1:**
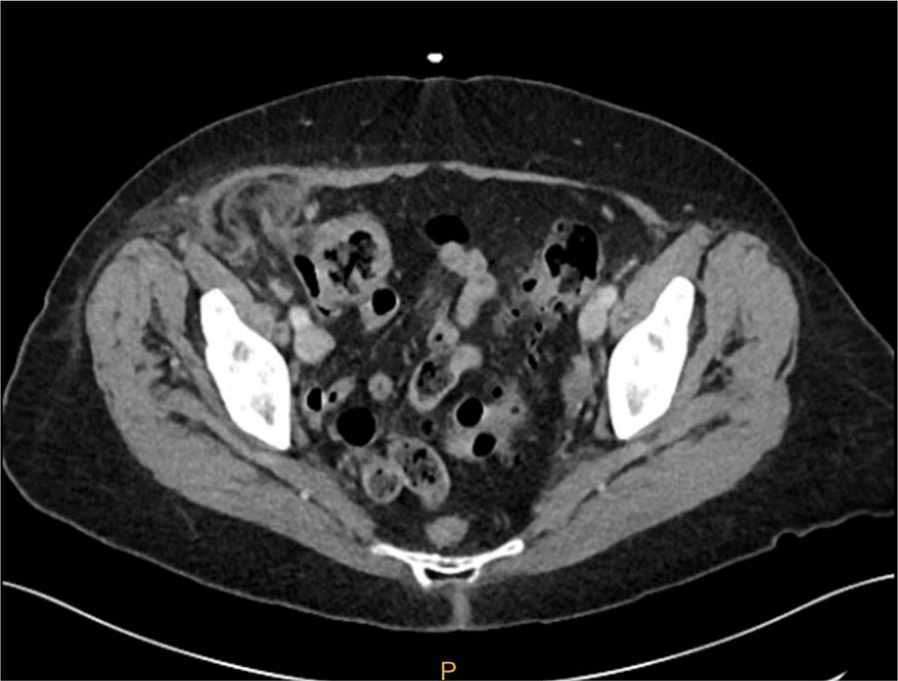
Preoperative CT image of right inguinal hernia. It is difficult from the images to determine if the hernia sac contains an appendix.

The patient was hemodynamically stable and afebrile in the emergency department. On examination, there was a palpable, non-reducible mass in her right lower abdomen that was tender to palpation. Remaining physical examination was unremarkable. Blood analysis of the patient demonstrated a normal WCC of 7.2 and an elevated CRP of 42. A CT abdomen and pelvis suggested there was a strangulated right ventral abdominal wall hernia lateral to the deep inguinal ring. The hernia contained the tip of the appendix with some free fluid and had associated fat stranding. The favoured provisional diagnosis was an incisional hernia containing her appendix due to her extensive previous abdominal surgery. Differential diagnoses included Amyand’s hernia (Fig. 1).

**Figure 2:**
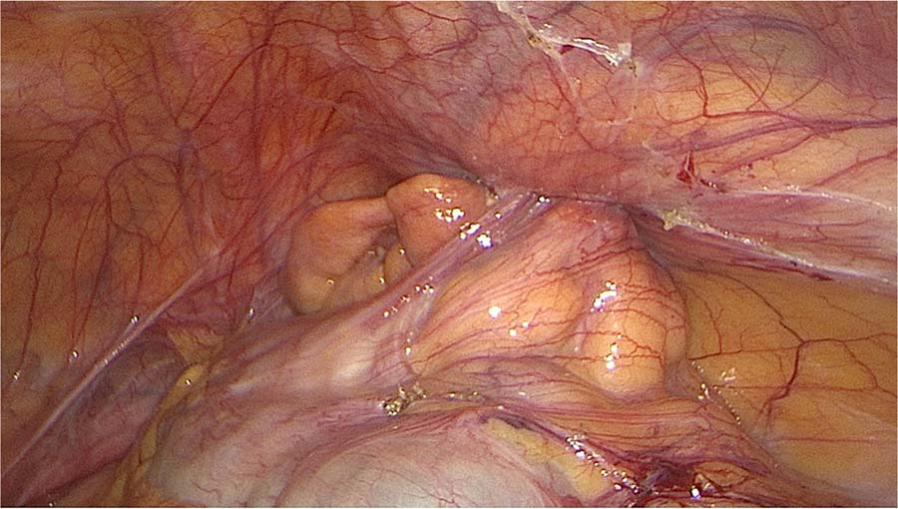
Intraoperative image of inflamed appendix within the internal inguinal ring.

The patient was taken to theatre and a diagnostic laparoscopy was performed. Visual examination intra-operatively demonstrated a right indirect inguinal hernia containing the appendix. The appendix was mildly inflamed and not perforated; however, the mesentery had an ischemic appearance (Fig. 2). Hernia contents were able to be reduced laparoscopically. Due to the appearance of the appendix and mesentery, an appendicectomy. Laparoscopic transabdominal preperitoneal primary repair of the hernia was performed. The patient was discharged from hospital the following day.

Histology showed an appendix with adjacent fat that demonstrated ischemic-like features with acute inflammation and fat necrosis. There was no evidence of dysplasia or malignancy. The patient was seen in clinic four weeks later and was recovering well.

## Discussion

The incidence of an Amyand’s hernia has been reported as 1% of inguinal hernia cases. Amyand’s hernia is more likely to occur in children than adults due to patency of the processus vaginalis [6]. In adults, an Amyand’s hernia is more common in men. Women presenting with an Amyand’s hernia were typically post-menopausal. Of those who present with an Amyand’s hernia, appendicitis has been reported in 0.1% of all cases [7].

The underlying mechanism that causes appendicitis in an Amyand’s hernia is not the same as true appendicitis. Obstruction of the appendix in an Amyand’s hernia is caused by extraluminal pressure of the hernia neck on the appendix rather than intraluminal obstruction [4, 8]. Incarceration of the appendix secondary to the hernia neck then leads to compromised blood flow, generalised inflammation, and bacterial overgrowth. The associated mesentery can be threatened through a similar mechanism [9].

Historically, surgical management of Amyand’s hernia was primarily through an open approach. A laparoscopic approach is becoming favoured due to patient benefits such as faster recovery, shorter hospital stays, and decreased post-operative pain [1, 10]. Visualisation of the appendix is an important guide for surgical management. Losanoff and Basson recommend different surgical approaches based upon appendix findings and associated abdominal pathology. For a normal appendix, they recommend a hernia repair with mesh repair. For acute appendicitis or other abdominal pathology, they recommend appendicectomy and a primary repair without mesh due to the risk of wound and mesh infection [11]. It is recommended that surgery be performed laparoscopically and only converted to a laparotomy if required [1].

We present a case report of a ventral abdominal hernia which was diagnosed intra-operatively as an Amyand’s hernia, which is a mildly inflamed appendix and ischemic-appearing mesentery. The Amyand’s hernia was managed successfully with a laparoscopic appendicectomy and transabdominal preperitoneal primary repair.
